# The pathogenic role of high-titer anti-Ro52 antibodies in neonatal lupus erythematosus: insights from a 9 case series

**DOI:** 10.3389/fped.2026.1939494

**Published:** 2026-10-07

**Authors:** Shuo Cheng, Yi Jiang, Suo Zhang, Jiaming Huang, Xiaoxue Wang, Huanyu Xie, Meiying Wang

**Affiliations:** 1 Department of Rheumatology and Immunology, Shenzhen Second People’s Hospital, The First Affiliated Hospital of Shenzhen University, Shenzhen, China; 2 Shenzhen University Medical School, Shenzhen University, Shenzhen, China

**Keywords:** anti-Ro52, autoimmune channelopathy, congenital heart block, lupus, neonatal lupus erythematosus

## Abstract

**Background:**

Neonatal Lupus Erythematosus (NLE) is a rare acquired autoimmune disease caused by the transplacental transfer of maternal anti-Ro/SSA and anti-La/SSB antibodies. Despite the high prevalence of these antibodies in mothers with Systemic Lupus Erythematosus (SLE) and Sjögren's Syndrome (SS), the clinical manifestations in neonates are highly heterogeneous, ranging from transient cutaneous lesions to life-threatening congenital heart block (CHB).

**Methods:**

This study analyzed a case series of nine mother-infant pairs diagnosed with NLE. We integrated clinical outcomes, maternal serological profiles (including quantitative anti-Ro52, Ro60, and La titers), and pharmacological interventions to explore the determinants of disease severity and organ-specific manifestations.

**Results:**

Among the nine mothers, six (66.7%) had Sjögren's syndrome and three (33.3%) had systemic lupus erythematosus, including two with secondary antiphospholipid syndrome. Notably, 88.9% of mothers were diagnosed with autoimmune disease during or after the affected pregnancy. All mothers demonstrated antinuclear antibody positivity and were uniformly positive for anti-SSA/Ro, anti-SSB/La, and anti-Ro52 antibodies. Cardiac involvement occurred in three pregnancies, including recurrent complete CHB leading to pregnancy termination in one mother. One fetus with second-degree atrioventricular block received prompt dexamethasone therapy and achieved successful reversal of conduction abnormalities. Cutaneous manifestations were observed in five neonates, while hematologic involvement, primarily thrombocytopenia and anemia, occurred in four cases. Maternal hypocomplementemia and elevated inflammatory markers appeared to be associated with more severe fetal outcomes.

**Conclusion:**

All nine mothers (100%) tested positive for high-titer anti-Ro52 antibodies, a finding consistent with the proposed role of anti-Ro52 as a risk biomarker for NLE. Intensive fetal echocardiographic surveillance between 16 and 26 weeks of gestation is vital to capture the critical window of low-degree conduction block, where synergistic immunomodulation with dexamethasone can reverse fetal heart block prior to irreversible nodal fibrosis. NLE serves as an early warning sign of occult maternal autoimmune disease, requiring long-term maternal follow-up.

## 1 Introduction

Neonatal Lupus Erythematosus (NLE) is a rare autoimmune syndrome resulting from the transplacental transfer of maternal autoantibodies to the fetus (1), associated with the presence of maternal anti-Ro/SS-A and anti-La/SS-B antibodies. Despite the high prevalence of anti-Ro/SSA and anti-La/SSB antibodies in patients with Systemic Lupus Erythematosus (SLE) and Sjögren's Syndrome (SS), the vast majority 98% of infants born to antibody-positive mothers remain free of severe complications such as cardiac conduction block (2), the incidence of NLE in susceptible pregnancies is approximately 2% (3). However, the recurrence risk rises significantly to 17%–20% for mothers with a history of a child affected by Congenital Heart Block (CHB) (4). Furthermore, NLE is the primary etiology of neonatal CHB, accounting for 80%–95% of cases diagnosed within the first 28 days of life (5, 6). Aside from cardiac manifestations, skin lesions are common in NLE, and the rate of skin involvement varies among different ethnic groups, with up to 80% of NLE patients in Asia having skin involvement (7, 8). The rash of neonatal lupus presents as transient annular erythematous scaling lesions on the face, scalp, trunk, and limbs, with histopathology demonstrating granular IgG deposition and vesicular changes at the dermal-epidermal junction and skin appendages (9). The rash usually appears after birth and is typically caused by sun exposure (10, 11), it has also been reported to correlate with anti-U1RNP antibodies in certain contexts (12). Interestingly, the clearance of these cutaneous manifestations closely mirrors the clearance of maternal anti-SSA/Ro antibodies from the infant's circulation (8). While historical research indicates that mothers of infants with NLE generally exhibit positivity for anti-SSA/Ro, anti-SSB/La, or anti-U1RNP antibodies (2), our study uniquely expands upon these observations by identifying anti-Ro52 antibodies as a distinct and highly uniform immunological signature within our cohort. Beyond these dermatological features, other transient systemic manifestations, including hepatobiliary disease and hematologic disorders, may manifest postpartum (13).

Our case study shows that mothers of neonatal lupus share common antibody characteristics, which can also affect the infant's heart, blood, skin, and other systems. We constructively present a case of a child with successfully reversed CHB, thus comprehensively reporting on NLE and gaining more potential experience for clinical work.

## 2 Methods

This retrospective, single-center case series was conducted at The First Affiliated Hospital of Shenzhen University between 2019 and 2025. Electronic medical records were systematically reviewed to identify mother-infant pairs affected by NLE. Antinuclear antibodies were detected by indirect immunofluorescence, and titers and patterns were recorded. Anti-Ro52 antibodies were quantified by enzyme-linked immunosorbent assay; results are expressed as the ratio of the sample optical density to the cut-off value (S/C.O.), with a ratio ≥1.0 considered positive. Anti-SSA/Ro, anti-SSB/La, anticardiolipin IgG, and anti-β2 glycoprotein I IgG antibodies were measured by the routine clinical laboratory assays used at our institution. Serum C3 and C4 were quantified by standard clinical laboratory methods and are expressed in g/L.

Patients were eligible if they had an NLE diagnosis established by a rheumatologist based on American College of Rheumatology criteria (14). Exclusion criteria comprised: (1) major congenital malformations, chromosomal anomalies, or inherited metabolic disorders; (2) critical data missingness that risked confounding results; and (3) lack of parental consent. The investigation protocol was conformed with the Helsinki Declaration and approved by the Ethics Committee of The First Affiliated Hospital of Shenzhen University.

Patient data were retrieved from electronic medical records. Extracted parameters included baseline perinatal demographics, familial/maternal autoimmune and allergic background, clinical presentations, laboratory profiles.

## 3 Presentation of cases

This study included nine mothers aged 28 to 38 years, who presented diverse histories of autoimmune diseases. Notably, SS dominated maternal diagnosis, consistent with literature reports that approximately 50% to 90% of mothers with NLE have SS or are in a subclinical state (13, 15).

### 3.1 Maternal demographic and clinical characteristics

Table 1 summarizes the demographic, clinical, and serological characteristics of the nine mothers included in this study. All mothers had a confirmed history of underlying autoimmune diseases. SS was the most prevalent diagnosis, observed in 6 of the 9 cases (66.7%). The remaining 3 patients (33.3%) were diagnosed with SLE, among whom two were concomitantly diagnosed with Antiphospholipid Syndrome (APS). In this cohort, 88.9% (8/9) of the mothers were diagnosed with autoimmune disease during or after this abnormal pregnancy. This underscores the absolute necessity for obstetricians to screen mothers for ANA and ENA profiles when encountering fetal arrhythmias or recurrent miscarriage.

**Table 1:** Demographic and clinical characteristics for mother.

Case	ANA	Karyotype	SSA	SSB	Ro-52	CL IgG	β2GP1 IgG	C3 (g/L)	C4 (g/L)	Diagnosis	Age at delivery	Age at diagnosis
1	1:3,200	Speckled/Nucleolar	2.63	3.34	3.75	0	0	1.48	0.18	SS	26	26
2	1:320	Speckled	1.95	3.43	2.04	1.9	2.1	1.29	0.269	SS	31	31
3	1:1,000	Speckled	2.26	0.86	3.14	1.95	2.92	0.83	0.14	SS	27	27
4	1:10,000	Homogeneous/Cytoplasmic	1.91	2.42	3.43	2.1	<2	0.87	0.173	SS	30	32
5	1:3,200	Speckled/Nucleolar	0.71	2.01	2.8	2.52	3.36	0.86	0.194	SLE	31	28
6	1:1,000	Unknow	+++	+++	+++	80.8	14.2	0.91	0.113	SLE/APS	29	29
7	1:1,000	Speckled	1.94	2.03	2.65	1,288	1,840	0.85	0.119	SLE/APS	25	25
8	1:1,000	Speckled	2.57	1.81	2.43	22.3	30	0.97	0.288	SS/APS	29	30
9	1:320	Speckled	2.07	1.45	3.95	4.2	2.5	1.02	0.106	SS	37	37

Immunological and serological evaluations revealed profound autoantibody reactivity in all subjects. As shown in Table 1, antinuclear antibodies (ANA) were universally positive (9/9, 100%), with titers ranging from 1:320 to 1:10,000. The speckled pattern was the predominant ANA karyotype. Notably, in contrast to previous studies reporting variable frequencies of anti-SSA/Ro, anti-SSB/La, and anti-Ro52 antibodies, all nine mothers in our cohort exhibited a remarkably uniform autoantibody profile, demonstrating triple positivity for anti-SSA/Ro, anti-SSB/La, and anti-Ro52 antibodies.

Antiphospholipid antibody screening showed that anticardiolipin (aCL) IgG and anti-β2 glycoprotein I (β2GP1) IgG levels were markedly elevated in the two patients with SLE and secondary APS (Cases 6 and 7), and mildly elevated in one SS patient (Case 8). Furthermore, serum complement levels varied among the cohort; borderline low or decreased levels of C3 and C4 were observed predominantly in patients with SLE/APS, reflecting potential complement consumption related to disease activity.

### 3.2 Cardiac manifestations of neonatal lupus

Cardiac involvement represented the most severe manifestation of NLE in this cohort, occurring in three pregnancies across two mothers (Cases 1, 2, and 9), all of whom carried high-titer anti-Ro-52 antibodies.

As shown in Table 2, Case 1 experienced two consecutive pregnancy losses attributable to fetal cardiac conduction block. During the first pregnancy, fetal echocardiography at approximately 16 weeks of gestation revealed a severe cardiac conduction block, resulting in an elective termination. Following a diagnosis of SS and the initiation of hydroxychloroquine, prednisone, and aspirin, the patient conceived again. At 20 weeks of gestation during her second pregnancy, fetal echocardiography confirmed a complete third-degree atrioventricular block complicated by severe fetal heart failure. Despite aggressive intervention with dexamethasoneand intravenous immunoglobulin (IVIG), fetal cardiac function could not be salvaged, necessitating a second termination. These outcomes underscore the irreversibility of CHB once fibrosis and calcification of the atrioventricular node are established. Case 2 whose SS diagnosis was incidentally prompted by the discovery of the fetal cardiac abnormality itself, presented with second-degree AVB detected at 26 weeks of gestation during routine monitoring. The rapid progression of the fetal cardiac lesion necessitated the termination of the pregnancy.

**Table 2:** Comprehensive maternal-fetal clinical characteristics and outcomes of the NLE case series.

Case	Neonatal		Maternal			Final outcome
	Cardiac involvement	Cutaneous manifestations	Hematological findings	Inflammatory & immunological markers	Gestational age	
1	2nd/3rd-degree AV block, severe heart failure	None	Anemia (Hb 89 g/L)	Elevated CRP (27.04 mg/L) High IgG (23.56 g/L)	20 weeks	Fetal demise
2	2nd-degree AV block	None	Normal	Stable	22 weeks	Fetal demise
3	None	Annular facial erythema	Anemia (Hb 116 g/L)	Stable	8 weeks	Live birth
4	None	Generalized rash	Normal	Stable	6 weeks	Live birth
5	None	Facial erythema	Normal	Stable	19 weeks	Live birth
6	None	Generalized rash	Anemia (Hb 102 g/L)	Elevated ESR (45 mm/h)	22 weeks	Live birth
7	None	Facial and chest erythema	Anemia (Hb 110 g/L) Leukopenia (WBC 3.5 × 10^9^/L)	Stable	6 weeks	Live birth
8	None	None	Normal	Stable	22 weeks	Live birth
9	2nd-degree AV block	None	Anemia (Hb 101 g/L)	Hypocomplementemia (Low C4: 0.106 g/L)	22 weeks	Ongoing

Case 9 provided the most instructive example of timely cardiac intervention in this cohort. At 22 weeks of gestation, intensive fetal echocardiographic surveillance detected the emergence of second-degree AVB. Recognizing this as a pathophysiological inflection point prior to irreversible atrioventricular nodal fibrosis, the clinical team promptly added dexamethasone (4 mg/day) to her existing regimen of aspirin and hydroxychloroquine.

### 3.3 Cutaneous involvement of NLE

Cutaneous involvement was the most frequently observed clinical manifestation among the live-born infants in Table 2, unambiguously identified in 5 cases (Cases 3, 4, 5, 6, and 7). The clinical presentation predominantly involved the facial region, though the extent of the lesions varied among the affected neonates.

According to the clinical data, Cases 3 and 5 presented primarily with isolated facial rashes. These typically manifest as classic periorbital erythema and annular erythematous plaques. Case 7 exhibited a more widespread dermatological phenotype, characterized by multiple red erythematous lesions distributed across both the facial region and the trunk. Cases 4 and 6 also presented with distinct neonatal rashes consistent with the NLE cutaneous profile. Morphologically, the typical NLE rash presents as annular or oval red patches with slightly raised edges and a central clearing, predominantly affecting sun-exposed areas such as the periorbital region, scalp, and trunk. These lesions typically emerge within the first few weeks of life and are highly photosensitive, often triggered or exacerbated by ultraviolet radiation exposure.

### 3.4 Hematologic involvement of NLE

Case 8 of neonatal thrombocytopenia highlighted hematologic involvement, with the infant's platelet count plummeting to 30 × 10^9^/L on the second day after birth. This is a typical hematologic presentation of NLE (16), due to increased platelet destruction caused by cross-reaction or immune complex formation between anti-Ro/La antibodies and fetal platelet antigens (17). Such thrombocytopenia is usually self-limiting, but if the titer is too low (<50), there is a risk of intracranial hemorrhage, requiring consideration of intravenous immunoglobulin (IVIG) intervention. In addition, several infants (cases 1, 3, 6, 7 and 9) had varying degrees of anemia, reflecting a temporary suppression of the bone marrow hematopoietic system.

### 3.5 The influence of maternal blood indicators on postpartum outcomes

The systemic inflammatory and immunologic burden of the mother directly dictates the severity of the placental and fetal microenvironment.

As shown in Table 2, case 1 had significantly elevated CRP (27.04 mg/L) and the lowest hemoglobin level (89 g/L), consistent with its worst pregnancy outcome. High inflammation levels may indicate high pathogenicity of antibodies or severe placental inflammation. Case 6 had an ESR of 45 mm/h, suggesting active SLE. Case 4, despite extremely high titers, had normal CRP and ESR, but high albumin levels (46.7 g/L), which may explain why the fetus had intact cardiac function despite the rash, suggesting that a lower overall maternal inflammatory burden provided some protection to the fetus.

Most patients had normal liver and kidney function, but weakly positive urine protein was observed in cases 1 and 3, which may suggest underlying mild lupus nephritis or renal burden during pregnancy. Complement consumption proved to be another critical biomarker. Serum complement levels varied among the cohort, with borderline low or significantly decreased levels of C3 and C4 observed predominantly in the patients diagnosed with SLE and secondary APS, reflecting profound complement activation and consumption related to disease activity at the maternal-fetal interface. Most alarmingly, Complement C4 reached its absolute nadir in Case 9 (0.106 g/L), a depletion that aligned with exquisite precision with the emergence of fetal cardiac conduction abnormalities. The activation of the complement system serves as a massive amplifier of tissue damage in NLE, and severe hypocomplementemia typically reflects the substantial tissue deposition and depletion of circulating immune complexes.

## 4 Discussion

Neonatal lupus is a classic model of passively acquired autoimmune disease, in which maternal IgG autoantibodies against the Ro/La ribonucleoprotein complex cross the placenta and target fetal tissues, causing immune-mediated damage to multiple target organs in the fetus and newborn (13, 18, 19). The clinical phenotypes of NLE are highly heterogeneous, ranging from well-prognostic, transient skin erythema to the most devastating and often irreversible autoimmune-mediated congenital heart block (20).

### 4.1 The core pathogenic role of anti-Ro52 antibodies

Within this cohort, the most striking immunological feature is the universal positivity of anti-Ro52 antibodies across all nine patients (100%), with the vast majority presenting at exceptionally high quantitative titers (values ranging between 2.04 and 3.95). In contrast, standard qualitative testing for anti-SSA/Ro and anti-SSB/La demonstrated slight variability.

Case 5 serves as a potent clinical warning, routine qualitative testing for anti-SSA/Ro antibodies yielded a negative result, yet the patient exhibited definitive positivity for anti-Ro52 antibodies (2.80) alongside anti-SSB/La. Her newborn subsequently developed classic facial cutaneous NLE. This observation aligns with the current immunological consensus that anti-Ro52 antibodies may play a role in fetal tissue injury, where higher quantitative titers are often associated with an increased likelihood and severity of CHB (21).

Our results are highly consistent with existing research, Salomonsson et al. showing that 95% of them had a positive anti-Ro52 antibody titer, and the frequency of Ro60 and La antibodies was 63% and 58%, respectively (22). Bouyon's study confirmed that the risk of fetal cardiac complications increases when the mother has anti-SS-A and anti-SS-B antibodies, especially when specific anti-Ro52 antibodies are detected. Meanwhile, the risk of neonatal lupus is lower in women with low antibody titers (23).

### 4.2 Mechanistic insights into Ro52 disruption and interferon signaling

Ro52, formally classified as tripartite-motif-containing protein 21(TRIM21), is an intracellular receptor and E3 ubiquitin ligase of the TRIM family (24, 25).

Maternal anti-Ro52 antibodies competitively block its ligase activity by binding the UBE2E1 site, causing IRF3/5/7 dysregulation, which triggers persistent type I interferon inflammation and upregulates Ro52 expression, forming a positive feedback loop of damage (26–28).

Anti-Ro52 antibodies, especially those targeting the p200 epitope, are more strongly associated with congenital atrioventricular block than anti-Ro60 or anti-SSB antibodies (21). After transplacental transfer, they cross-react with fetal cardiomyocyte calcium channels, causing calcium dyshomeostasis, overload, and apoptosis (29, 30). Instead of silent clearance, maternal antibodies drive macrophage-mediated inflammatory phagocytosis, releasing abundant TNF-α and TGF-β, leading to atrioventricular node fibrosis, calcification, and permanent third-degree block (29, 31).

In this cohort, recurrent CHB (Case 1) and new-onset AVB (Case 9) were both linked to high anti-Ro52 antibody levels.

The three cardiac cases illustrate CHB's polarized prognosis. Although the baseline CHB risk in anti-Ro-positive primiparous women without prior adverse history is only 1%–2%, a previous infant with CHB or cutaneous NLE raises the recurrence risk to 17%–25%, with increased likelihood of cardiomyopathy and heart failure (32, 33).

### 4.3 Inspiration from HCQ and hormones for NLE

Eight mothers in our 9-case cohort were treated with HCQ during gestation. Biological models suggest that HCQ disrupts TLR-7/9 endosomal activation and downregulates pro-fibrotic signaling in macrophages (34, 35). Previous studies have associated maternal HCQ use with decreased CHB recurrence and favorable obstetric outcomes, consistent with the predominantly mild cutaneous or transient presentations observed in our infants. In this cohort, newborns of patients who took HCQ long-term mostly showed only mild skin lesions, and some intrauterine arrhythmias were reversed (36, 37).

The successful reversal of second-degree atrioventricular block in case 9 is the most important finding of this cohort. Third-degree CHB is usually irreversible due to fibrosis, while low-degree conduction block is in an active inflammatory stage and can respond to fluorinated glucocorticoids. Dexamethasone (4 mg/day) successfully suppressed fetal myocardial inflammation and prevented its progression to irreversible CHB. These observations call for routine fetal echocardiographic surveillance every 1–2 weeks between 16 and 26 weeks of gestation for high-risk pregnancies.

### 4.4 Multiple anticoagulation and anti-inflammatory strategies for mothers with NLE complicated by APS

Both patients had SLE complicated by APS. In addition to being positive for anti-SSA/SSB, their aCL IgG and β2GP1 were significantly elevated, indicating an extremely high risk of placental microthrombosis and a high risk of intrauterine fetal death or severe preeclampsia. Both patients received triple therapy with aspirin (100 mg/day), low molecular weight heparin (0.4 mL/day), and hydroxychloroquine, and both delivered successfully. Approximately 50% of mothers of infants with NLE are completely asymptomatic at delivery (38), and their high-titer antibodies are only discovered after neonatal diagnosis. However, long-term follow-up shows that more than half of asymptomatic mothers gradually develop autoimmune diseases several to more than ten years postpartum, most commonly SS, followed by SLE (39, 40).

In this cohort, the diagnosis time of SS in several mothers highly coincided with their pregnancy events, suggesting that NLE may be an early warning sign of occult maternal autoimmune diseases. Therefore, the experience of giving birth to a child with NLE should be regarded as a long-term warning of maternal health and requires continuous follow-up.

## 5 Conclusion

Maternal anti-Ro52 autoantibodies, particularly targeting the p200 epitope, are associated with an increased risk of NLE and multi-system organ involvement, especially fetal myocardial injury. While HCQ remains a key therapeutic option in high risk pregnancies, carefully timed immunomodulatory strategies warrant further exploration to evaluate their potential in mitigating conduction abnormalities before irreversible fibrosis develops. Given the small sample size, future prospective, multi-center studies are needed.

## Data Availability

The raw data supporting the conclusions of this article will be made available by the authors, without undue reservation.
